# Yellow nail syndrome resulting from cardiac mitral valve replacement

**DOI:** 10.1186/s13019-019-0903-1

**Published:** 2019-04-11

**Authors:** Hossein Sarmast, Ahmad Takriti

**Affiliations:** 1 Cardiovascular surgery in cardiac surgery hospital of Damascus university, Mouasat Square, Omar ben Abdulaziz Street, Damascus, Syria; 2 Department in cardiac surgery hospital of Damascus university, Damascus, Syria

**Keywords:** Yellow nail syndrome, Mitral valve replacement, Thoracic duct injury, Chylothorax, Pleurodesis, Pleural effusion, Total parentral nutrition, lymphedema

## Abstract

**Background:**

Yellow nail syndrome is a rare disease with unknown etiology, Attributed to functional anomalies or disturbance in lymphatic drainage. This condition is characterized by triad of nail discoloration, respiratory or intrathoracic manifestations and lymphedema.

**Case presentation:**

Twenty days after mitral valve replacement for severe rheumatic mitral valve stenosis, 39 years old woman presented with face tenderness and hearing problems besides stuffy and clogged nose and underwent routin rhinosinusitis therapy. She came back to ears, nose and throat service with persistent rhinosinusitis as well as relapsing preoperative couphs and dyspnea besides lower extremities edema and toenails discoloration. After some modulations of treatment, she was introduced to pulmonary clinic on post -operative day = 30. Chest x ray showed a lot of left pleural effusion then she was returned to our service (cardiac surgery) on post- operative day = 33. The pigtail catheter was secured and we attained a significant amount of milky fluid which conformed with chylothorax. Finally Yellow nail syndrome was diagnosed with her on post–operative day = 35. Early conservative therapy such as bed rest, legs massage, low fat diet with medium chain triglycerides, diuretics, bronchodilator inhaler was not be able to satisfy us (chylous out put > 330 cc/d). Therefore the catheter replacement with chest tube was carried out followed by pleurodesis using Talc and doxycycline besides transition of oral intake to total parentral nutrition and vitamine E supplement, on post - operative day = 41. After that chylous leakage gradually subsided and patient was discharged to home on post- operative day = 47. At 4 weeks follow ups, chest x ray was clear without effusion and nails discoloration and legs lymphedema resolved.

**Conclusion:**

We reported the third post cardiac surgery Yellow nail syndrome which is an unclear entity with a set of associated signs and symptoms. Two prior reports involved with coronary artery bypass graft whereas we performed mitral valve replacement. In angiogram thoracic duct was not identified so that it seems post cardiac surgery Yellow nail the syndrome has iatrogenic origin due to the thoracic duct or its tributaries injury and requires meticulous assessment and management.

## Background

Yellow nail syndrome (YNS) is an idiopathic rare condition attributed to functional abnormalities of lymphatic drainage. It is characterized by three cardinal signs related to yellow nails, lymphedema and respiratory manifestations [1]. That yellowing represents a subset of chromonychia, defined as pathological nail discoloration, especially xanthonychia (yellow nail coloration). It is a syndrome that associated with conditions as different as diseases implicating the lymphatic system, autoimmune diseases or cancers [2, 3]. The first case of YNS was probably reported by Heller in 1927 [4], but Samman and White described the first series of patients with YNS accompanied by lymphedema in 1964 [5]. Whose report consisted of 13 patients whom had slow measured nail growth associated with nail discoloration ranging from pale yellow to dark greenish [6]. According to the information comes from a databases called HPO, the most manifestations that may be in as high as 80–99% of patients are: Bronchiectasis, nail dysplasia, lymphatic vessels hypoplasia, lymphedema and yellow nails followed by couph, dyspnea, pleuritis, RRI and rhinosinusitis in 30 -79% of patients [7, 8]. Emerson added pleural effusion to the diagnostic criteria [9]. Although two criteria from first group are required to diagnosis, it is difficult to call the entity YNS without nail discoloration. In addition to being yellow, nails may lack cuticle [10, 11], grow very slowly and become detached (onycholysis) [12–14]. Respiratory problems include chronic couph, bronchiectasis and pleural effusion. The complete Triad is present only in 27–69% of patients [15–17]. YNS often occurs in adults (age > 50 years) with no sex predominance [18]. Estimated prevalence is < 1/10000000 [19, 20]. YNS in pediatrics is very rare [21, 22].

### Case presentation

**Fig. 1:**
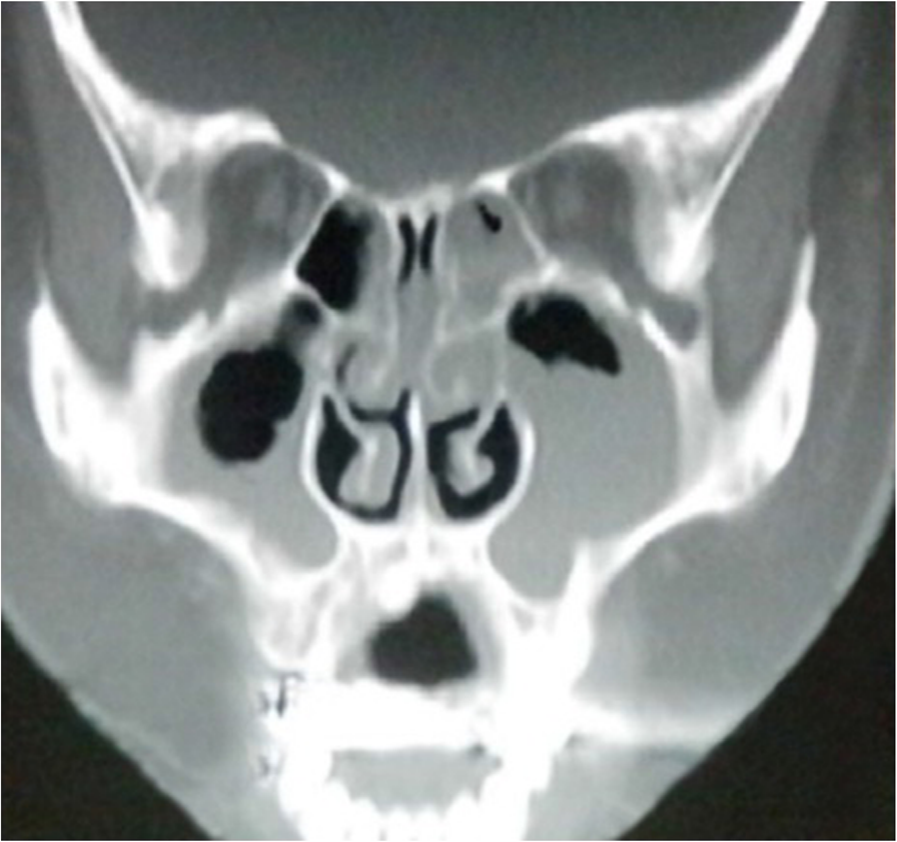
Sinuses computed tomographic scan of 39 years old woman after cardiac surgery. Sinuses imaging showed acute rhinosinusitis & homogenous parasinuses densities 20 days after miral valve replacement

**Fig. 2:**
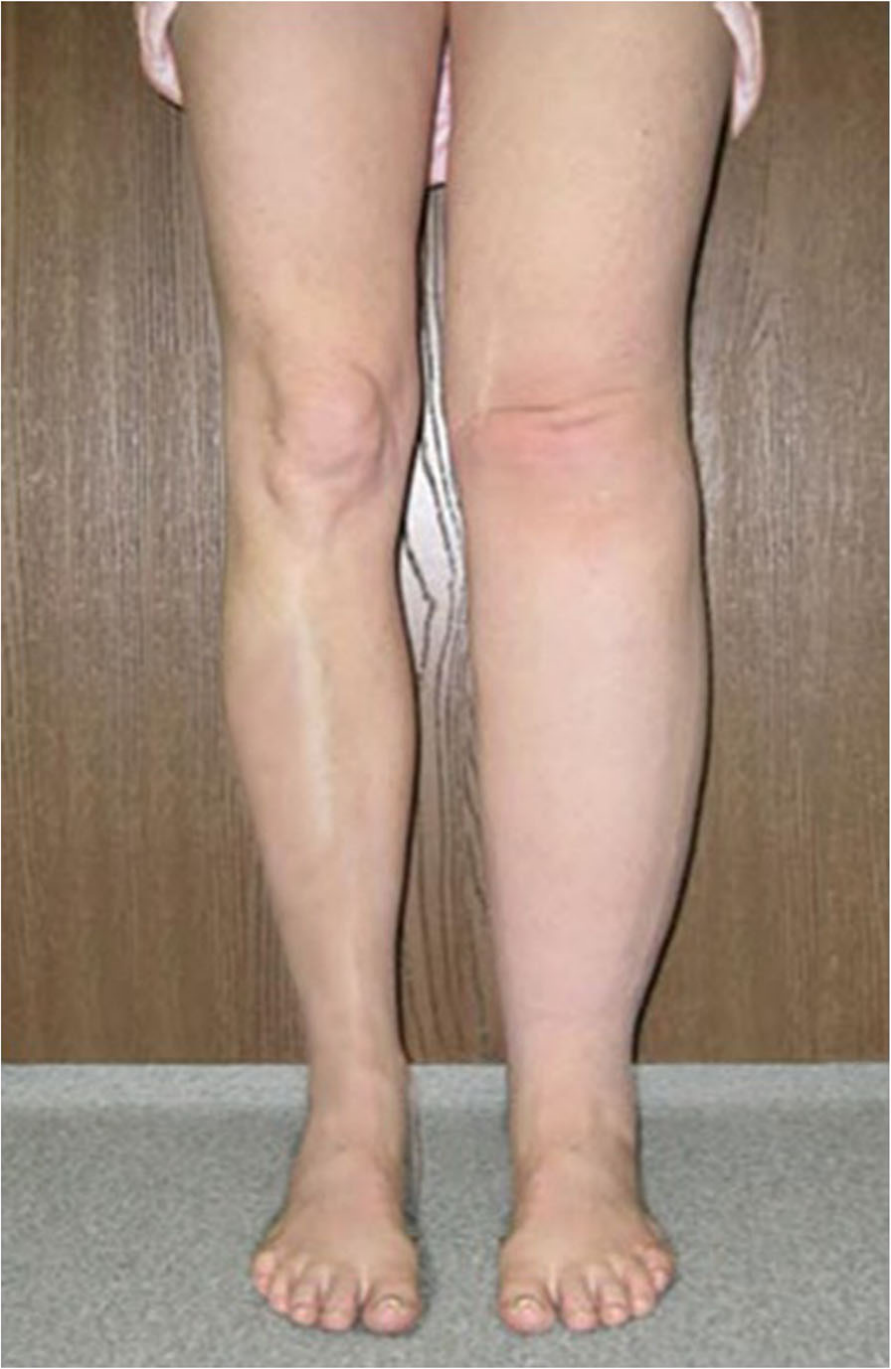
Lower extremities photograph of 39 years old woman after cardiac surgery. Photograph of legs showed bilateral lymphedema (specially in the left side) conforms to Campisi clinical stage two degree, 30 days after miral valve replacement

**Fig. 3:**
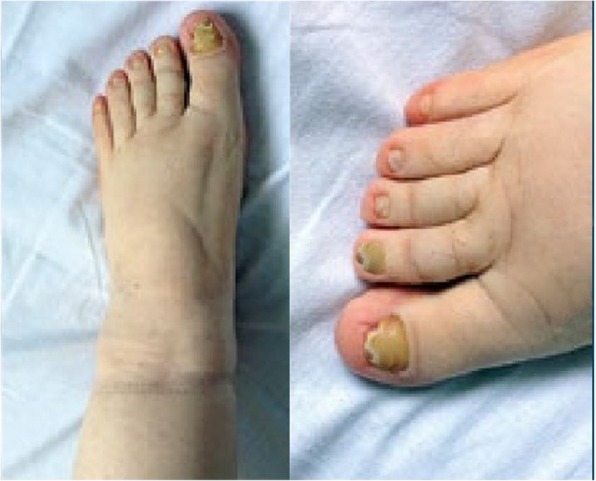
Yellow toenails discoloration in 39 years old woman after cardiac surgery. 30 ays after cardiac mitral valve replacement, toenails began to yellowish discoloration, onycholysis, hyperkeratosis and disappearance of the Lunula and Cuticle

**Fig. 4:**
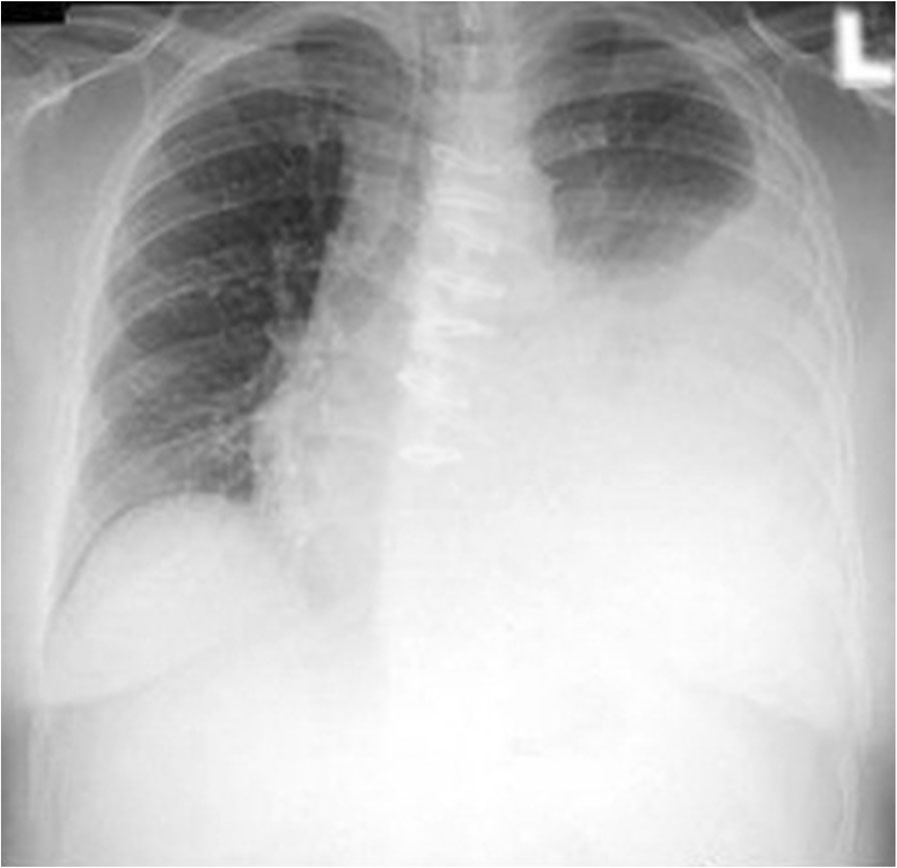
Pleural effusion detected by Chest X ray in 39 years old woman after cardiac surgery. Chest imaging showed a larg amount of left pleural cavity effusion, 30 days replacement after miral valve

A 39- years old multiparous woman has suffered from mild MS for 10 years, as a result of childhood bacterial tonsilopharyngitis (rheumatic MS). The first attacks of symptoms appeared in her last year of second decade, during her second pregnancy. Her chief complaint was exertional dyspnea. Two - dimensional echocardiography (2D-ECG) revealed a decreased mitral valve area (1.5 cm2). She had no other medical conditions and her past medical history included nothing else of note. Recently the symptoms worsened and she began to experience dyspnea in ordinary activities, AF and hemoptesia (hemoptysis). 2D-ECG positive findings were: critical diminished mitral valve area (0.8 cm2), left atrial enlargement without any organized clot attachment, thickened leaflets, commissural and subvalvular fussions and mean diastolic pressure gradient across valve =12 mmHg. She was scheduled for MVR with mechanical prosthesis. Open cardiac surgery with CPB using bicaval connulation was carried out. Early post-operative course was uneventful and she was discharged to home on POD = 7 with warfarin prescription without any prohibition about taking it, since she had completed her family members. She felt face tenderness and hearing problems besides stuffy and clogged nose on POD = 20. She underwent ENT consultation and based on the clinical and paraclinical finding, met ARS diagnosis (Fig. 1). As soon as an antihistamine, anticongestion and macrolide antibiotic was initiated. She came back to ENT service with persistent ARS as well as relapsing preoperative couphs and dyspnea besides lower extremities edema (Fig. 2) and toenails discoloration (Fig. 3). After some modulations of therapeutic drugs, she was referred to pulmonary clinic on POD = 30. CXR confirmed a significant left pleural effusion (Fig. 4) and she was readmitted in our service while taking lasix and SABAs (albuterol inhaler) in addition to aforementioned medicines on POD = 33. Immediately a pigtail catheter was secured with the purpose of both assessment and management. At first the fluid was bloody then became milky. Finally we attained 1850 cc frankly white fluid. Bedside ether test revealed the presence of fat which was confirmed by lab study (triglycerides = 1750 mg/dl chylomicron). We encountered with a patient who had different signs such as chylothorax, lymphedema and yellow discoloration of toenails. After dermatologist consultation we arrived at Yellow Nail Syndrome on POD = 35. Immediately conservative treatment was taken place such as bed rest, massage and compression therapy of legs using garments, restricted diet consist of high protein low fat with medium chain triglycerides and octreotide. Since the decline of chylous drainage was unsatisfied (330 cc/d), we exchanged the pigtail catheter with an appropriate size chest tube and TPN was initiated. Then talc and doxycycline pleurodesis was carried out besides Vitamine E supplement on POD = 41. Fortunately chest tube output substantially decreased, allowing transition to low fat oral intake diet on POD =45. Chest tube was removed and the patient was discharged on POD = 47. At 4 weeks follow ups, CXR was clear without effusion and nails discoloration and legs lymphedema resolved.

## Discussion and conclusions

**Fig. 5:**
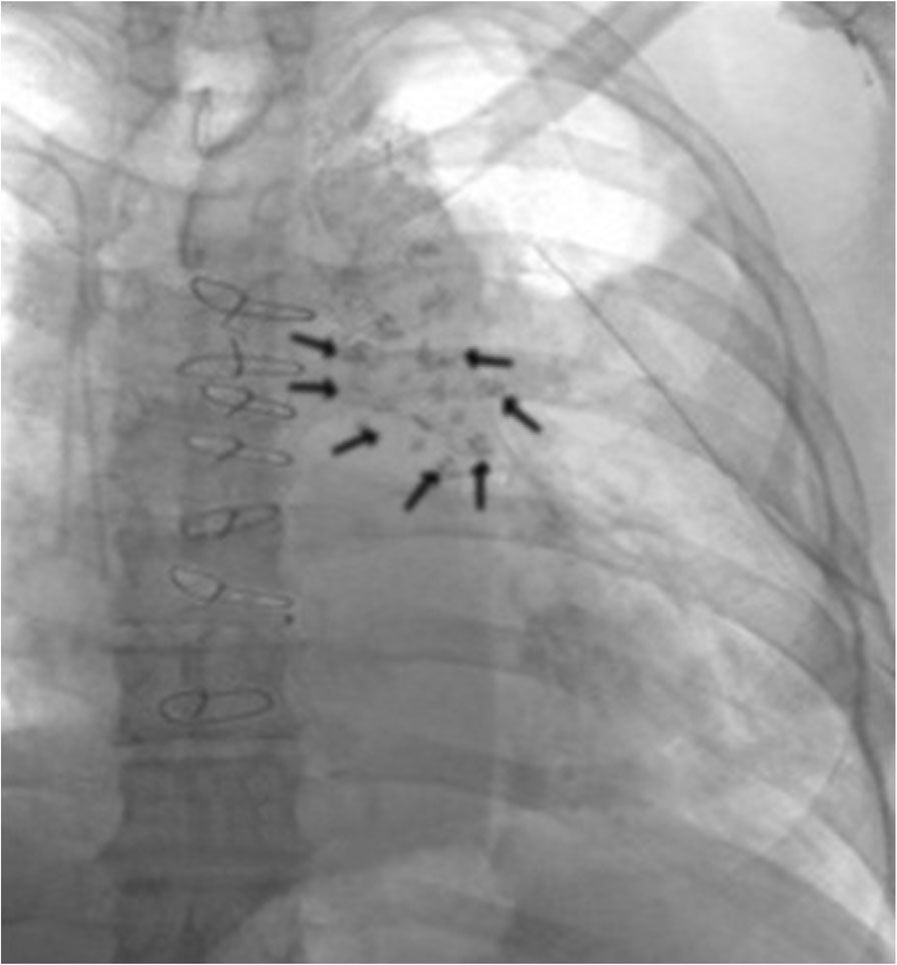
Lipidol droplets clarification by lymphangiogram for assessment the ethiology of post cardiac surgery pleural effusion in 39 years old woman. Lymphangiogram Demonstrated free contrast patches in the mediastinum without detecting of cycterna chili and thoracic duct, 41 days after cardiac mitral valve replacement

YNS has remained vague entity owing to lack of information about this very rare condition.There are little available data determining exact prevalence of YNS, as fewer than 400 cases have been published in the literature. Although two instances of familial cases have already been reported that in one sample, two siblings and in the other example, four offsprings during two generations had YNS [23, 24], the very few reported familial cases mimic a dominant inheritance pattern, which is not supported by any genetic evidence. In the other words, it has not been suggested any genetic link [25, 26]. It often occurs on its own even though is occasionally associated with autoimmune disease [27], lymphatic disease or cancers immunodeficiency disorders, such as common variable immunodeficiency, combined T- and B-cell deficiency [28, 29], Guillain–Barré syndrome [30], nephritic syndrome [31, 32], Hashimoto’s thyroiditis, severe hypothyroidism or hyperthyroidism [33, 34], xanthogranulomatous pyelonephritis [35] and rheumatoid arthritis even without thiol-analog use [36]. Very rare ocular involvement has been reported: chemosis, corneal micropannus (vascularized sheet of fibroustissue overlying the cornea), eyelid lymphedema, thickened conjunctiva [37]. Anecdotal associations have also been described: anhydrosis, pectus excavatum, eosinophilia–myalgia syndrome, bullous stomatitis, sarcoidosis and Raynaud’s phenomenon, cerebralaneurysm and pancytopenia. YNS is very rarely associated with primary intestinal lymphangiectasia (Waldmann’s disease) (OMIM 152800, ORPHA90362) or lymphedema – distichiasis syndrome (OMIM 153400, ORPHA33001), suggesting that these entities have overlapping characteristics, including lymphatic impairment [38, 39]. YNS treatment is not codified. YNS may resolve in few months without treatment [40] or, when it is a paraneoplastic syndrome, after cancer therapy [41]. Symptomatic treatments are prescribed. Patients may receive antibiotics for acute exacerbation of bronchiectasia, whereas, for patients with poor symptom control and/orrecurrent exacerbations, low-dose antibiotic prophylaxis, such as oral azithromycin (usually 250 mg 3 times/week), achieved attenuation of chest symptoms for the majority of them. Physiotherapy training (postural drainage, chest physiotherapy, flutter valve), combined or not with antibiotic prophylaxis, is also prescribed to help patients self-manage their chronic expectoration. Vaccinations against flu and pneumococci are strongly recommended. Surgical intervention of recurrent and/or large pleural effusions is useful: decortication/pleurectomy, pleurodesis (talc, picibanil quinacrine) and pleural–peritoneal shunts were the most effective treatments of symptomatic pleural effusions with, respectively, 89, 82 and 67% partial or complete responses [42, 43]. Octreotide, a somatostatin analog, was also used to treat YNS pleural effusions or chylous ascites and lymphedema, and generated positive responses [44–47]. We reported the third post cardiac surgery YNS. The first was reported in 2009 and an 80 years old male presented with YNS after CABG. The second report in 2018, belongs to a 62 years old man who endured CABG too and after that suffered from YNS. In both of them, there was coexistence regarding the LIMA harvesting and chylothorax (involving 81%), suspiciously owing to tight proximity between LIMA and thoracic duct. How is about our case? Our operation nowise correlated with LIMA. We don’t have any information about patient lymphatic system prior to the operation whether it was normal or aberrantly abnormal [48–52]. In our case the lymphangiogram demonstrated diffused abnormal indocyanine pattern in lower extremities especially on the left side but was not be able to identify the cycterna chyli and thoracic duct while showed sparse droplets of lipidol in left pleural cavity without any apparent leakage site (Fig. 5). On the other hand, doppler echocardiography precluded possibility of central venous embolization. All of the aforementioned evident findings are saying that, damage to the thoracic duct or lymphatic vessels is undertaken unless proven otherwise. According to the observations in majority of series, post thoracotomic chylothorax has been reported in the frequency of 0.2% [52–57]. Blalock and coworkers for the first time noted chylothorax following SVC ligation in 1936 . Then other surgeons observed similar findings (chylothrax) after surgical corrections such as: aortic coarctation, BTS, vascular slings, PDA, TOF, TS, VSD and APVC. Nobody other than two authors has reported full blown post cardiac surgery YNS and our report is third.

## Abbreviations

2D-ECG: Two dimensional echocardiography
APVC: Anomalous pulmonary venous
ARS: Acute rhinosinusitis
BTS: Blalock-taussing shunt
CABG: Coronary artery bypass grafting connection
CXR: Chest X ray
ENT: Ears, nose and throat
HPO: Human phenotype ontology
LIMA: Left internal mammary
MS: Mitral stenosis
MVR: Mitral valve replacement
PDA: Patent ductus arteriosus
POD: Post–operative day
RRI: Recurrent respiratory infection
SABAs: Short – acting beta agonists
SVC: Superior vena cava
TOF: Tetralogy of fallot
TS: Tricuspid stenosis
VSD: Ventricular septal defect
YNS: Yellow nail syndrome
